# Hemiarhinia: a rare presentation of the unilateral absence of nasal structure

**DOI:** 10.11604/pamj.2022.42.166.35225

**Published:** 2022-06-30

**Authors:** Divya Ramamoorthy, Sachin Damke

**Affiliations:** 1 Department of Pediatrics, Jawaharlal Nehru Medical College, Datta Meghe Institute of Medical Sciences, Sawangi, Wardha, Maharashtra, India

**Keywords:** Hemiarhinia, malformation, nasal

## Image in medicine

Hemiarhinia is a rare congenital malformation of unknown aetiology, unilateral nasal aplasia that includes the absence of an external nose and internal nasal cavity. Around 75 cases have been reported to date. Here we presented a case of 2 years child who comes to the outpatient department with complaints of nose deformity since birth. There were no associated symptoms with the deformity. There were no associated anomalies. The child was mentally normal, and their growth was within normal limits.

**Figure 1:**
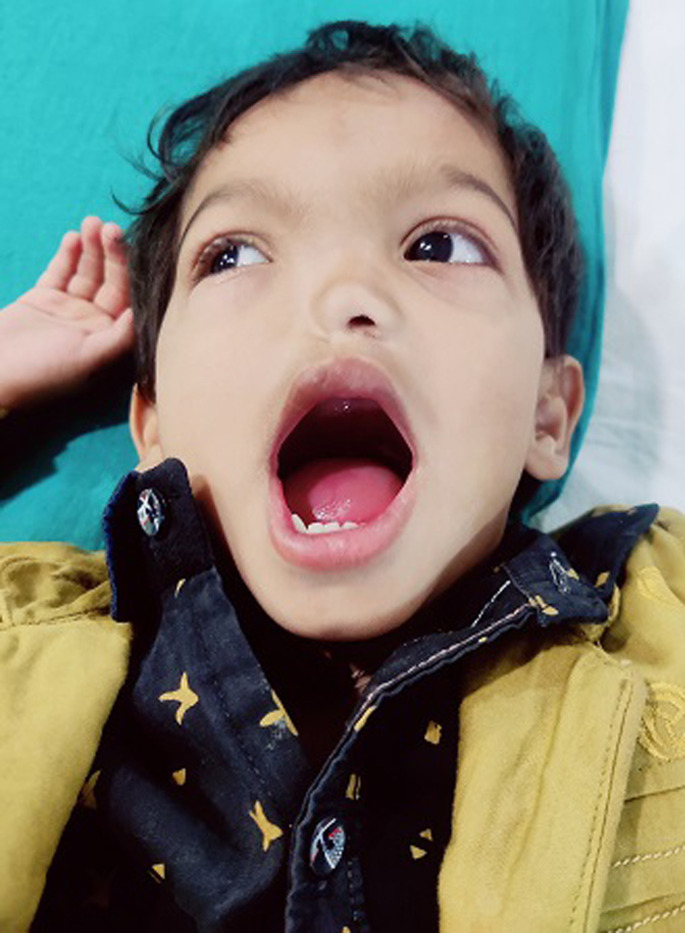
absence of nasal structure, including internal nasal cavity and external nose

